# Fermented grape seed meal promotes broiler growth and reduces abdominal fat deposition through intestinal microorganisms

**DOI:** 10.3389/fmicb.2022.994033

**Published:** 2022-10-10

**Authors:** Shanshan Nan, Min Yao, Xiaoyang Zhang, Hailiang Wang, Jiacheng Li, Junli Niu, Cheng Chen, Wenju Zhang, Cunxi Nie

**Affiliations:** ^1^College of Animal Science and Technology, Shihezi University, Shihezi, China; ^2^School of Medicine, Shihezi University, Shihezi, China

**Keywords:** fermented grape seed meal, yellow feather broilers, gut microbiota, growth performance, abdominal fat deposition

## Abstract

The fermentation of grape seed meal, a non-conventional feed resource, improves its conventional nutritional composition, promotes the growth and development of livestock and fat metabolism by influencing the structure and diversity of intestinal bacteria. In this study, the nutritional components of Fermented grape seed meal (FGSM) and their effects on the growth performance, carcass quality, serum biochemistry, and intestinal bacteria of yellow feather broilers were investigated. A total of 240 male 14-day-old yellow-feathered broilers were randomly selected and divided into four groups, with three replicates of 20 chickens each. Animals were fed diets containing 0% (Group I), 2% (Group II), 4% (Group III), or 6% (Group IV) FGSM until they were 56 days old. The results showed that Acid soluble protein (ASP) and Crude protein (CP) contents increased, Acid detergent fiber (ADF) and Neutral detergent fiber (NDF) contents decreased, and free amino acid content increased in the FGSM group. The non-targeted metabolome identified 29 differential metabolites in FGSM, including organic acids, polyunsaturated fatty acids, and monosaccharides. During the entire trial period, Average daily gain (ADG) increased and Feed conversion ratio (FCR) decreased in response to dietary FGSM supplementation (*p* < 0.05). TP content in the serum increased and BUN content decreased in groups III and IV (*p* < 0.05). Simultaneously, the serum TG content in group III and the abdominal fat rate in group IV were significantly reduced (*p* < 0.05). The results of gut microbiota analysis showed that FGSM could significantly increase the Shannon and Simpson indices of broilers (35 days). Reducing the relative abundance of *Bacteroidetes* significantly altered cecal microbiota composition by increasing the relative abundance of *Firmicutes* (*p* < 0.05). By day 56, butyric acid content increased in the cecal samples from Group III (*p* < 0.05). In addition, Spearman’s correlation analysis revealed a strong correlation between broiler growth performance, abdominal fat percentage, SCFAs, and gut microbes. In summary, the addition of appropriate levels of FGSM to rations improved broiler growth performance and reduced fat deposition by regulating gut microbes through differential metabolites and affecting the microbiota structure and SCFA content of the gut.

## Introduction

Grapes are the largest fruit crop in the world, and 80% of grape production is used for wine making (Yang and Xiao, 2013). A large amount of grape seed by-products are formed during the winemaking process that are not properly exploited and utilized. Recently, there has been increasing concern about reducing the impact of agro-industrial waste through its use as a new feed product (Câmara et al., 2020). Grape seeds have been found to be rich in fatty acids, amino acids, dietary fiber, polysaccharides and other nutrients (Zhu et al., 2015). It contains a large number of bioactive substances (such as proanthocyanidins, resveratrol and tannins, etc.) and is a feed material with good functional properties and biological activity (Chikwanha et al., 2018; Tang et al., 2018). Therefore, the development of grape seed meal resources combined with modern biotechnology not only strengthens the contribution to the implementation of circular economy, but also alleviates the current situation of feed resource shortage.

Feed biological pre-digestion techniques mainly include fermentation and enzymatic hydrolysis. Through microbial metabolic activities, feeds are enriched with more probiotics, metabolites, organic acids and other small molecules (Yang et al., 2021). After the livestock consumes the fermented feed, the number of probiotic bacteria in the feed increases and the pH value in the intestine decreases. Thus, this inhibits the growth of harmful bacteria and achieves a balance of bacteria in animal intestines (Heres et al., 2003; Chen and Yu, 2020). Consumption of fermented feed can also improve immunity (Abu-Elala et al., 2020), reduce antioxidative stress (Wu et al., 2015), increase feed intake and improve growth performance among other effects (Zhu et al., 2020). In addition, enzymatic hydrolysis was used to degrade anti- nutritional factors and increase small peptides in raw materials (Tie et al., 2020). Following feeding, animals are deficient in endogenous enzyme supplementation (Engberg et al., 2004; Alagawany et al., 2018), improving intestinal villus growth (Ritz et al., 1995; Berwanger et al., 2017) and improving nutrient uptake capacity (Córdova-Noboa et al., 2020).

Fermented Feed by Bacteria Coupled Fermentation with Enzymes has the advantages of both of these, rich in a large number of probiotics, exogenous enzymes and metabolites. Because the cecum of chickens has a large number of microorganisms and is abundant in species, they interact closely and densely with the host and ingested feed. Moreover, there is growing evidence that manipulating the composition of the gut microbiota through diet can alter the metabolic pathways and metabolite production (e.g., SCFAs) of the host, ultimately having a significant impact on the themself (Sugiharto and Ranjitkar, 2019). Therefore, according to its key functions, we hypothesized that the nutritional composition of grape seed meal would change after fermentation, producing metabolites that may have beneficial effects on broiler growth performance and intestinal health. In this study, based on untargeted metabolomics to investigate the effect of feed predigestion technology on the nutritional composition of grape seed meal, and studied the effects of different levels of FGSM added to the basal diet on the growth performance, slaughter performance, serum biochemistry, intestinal bacteria, and SCFAs content of broilers.

## Materials and methods

All experimental procedures were performed in strict accordance with the guidelines and were reviewed and approved by the Institutional Animal Bioethics Committee of Shihezi University (Xinjiang, China).

### Materials

Grape seed meal was provided by Western Animal Husbandry Co., Ltd., *Saccharomyces cerevisiae* was preserved by the Feed Biotechnology Laboratory of Shihezi University, and acid protease was purchased from Shanghai Yuanye Biotechnology Co., Ltd. Other feed ingredients, such as soybean meal and corn, were purchased from the Shihezi Farmers’ Market in Xinjiang. FGSM was fermented using *Saccharomyces cerevisiae* and acid protease enzymes in the Feed Biotechnology Laboratory of Shihezi University. UGSM was used as the control group (CON) without protease and probiotics were added for fermentation.

### Preparation of FGSM

The fermentation process of grape seed meal was as follows: the mixture ratio of grape seed meal, corn flour, and bran was 90:5:5, and the ratio of substrate to water was 1:0.8. The mixed substrate was then autoclaved at 121°C for 15 min. After high-pressure cooling, 60 ml (bacterial solution concentration of 2.97 × 10^9^ CFU/ml) of *Saccharomyces cerevisiae* liquid and 120 μg/g of acid protease (dissolved in double-distilled water before use and filtered with a 0.22 μm filter membrane for sterilization) were inoculated into each kg of fermentation substrate. Consistent mixing was performed followed by 48 h of fermentation in an incubator at 30°C. At the end of fermentation, the samples were dried in an oven at 45°C and pulverized through a 60-mesh sieve for storage. Finally, the dried samples were ground and stored at room temperature until mixture into experimental rations.

### Routine nutrient testing of FGSM

Dry matter (DM), crude protein (CP), ether extract (EE), Ca, P, ASH, and acid-soluble protein (ASP) content in FGSM were determined according to the AOAC (Association of Official Analytical Chemists) (2000) method. The neutral detergent fiber (NDF) and acid detergent fiber (ADF) contents were determined using the thermostable amylase method and its residual ash method (Van Soest et al., 1991). Free amino acid profile analysis was performed according to a previously reported method (Liu et al., 2018).

### Untargeted metabolomics analysis of FGSM

#### Metabolite extraction

100 mg of samples were weighed, precooled extract (methanol:acetonitrile:water volume ratio: 2:2:1) was added, and the solution was vortexed. Low-temperature sonication was performed on the samples for 30 min, then samples were incubated at −20°C for 10 min. Samples were centrifuged at 14,000*× g* for 20 min at 4°C, and the supernatant was collected for vacuum drying. During mass spectrometry, 100 μl of acetonitrile aqueous solution (acetonitrile:water volume ratio of 1:1) was added for rehydration. Samples were vortexed then centrifuged at 14,000*× g* for 15 min at 4°C, and the supernatant was analyzed.

#### Chromatographic conditions

An Agilent 1290 Infinity LC Ultra High-Performance Liquid Chromatography (UHPLC) HILIC column was used to separate samples; column temperature was 25°C; flow rate was 0.5 ml/min; mobile phase A was comprised of water +25 mm ammonium acetate +25 mm ammonia, and mobile phase B was acetonitrile with gradient elution. The procedure was as follows: 0 ~ 0.5 min, 95% B; 0.5 ~ 7 min, B varied linearly from 95 to 65%; 7 ~ 8 min, B varied linearly from 65 to 40%; 8 ~ 9 min, B was maintained at 40%; 9 ~ 9.1 min, B varied linearly from 40 to 95%; 9.1 ~ 12 min, B was maintained at 95%; the sample was placed in the autosampler at 4°C during the whole analysis. 4°C autosampler. The samples were also analyzed in random order to minimize the effect of fluctuations in the instrument signal. QC samples were inserted in the sample queue to monitor and evaluate the stability of the system and the reliability of the experimental data.

#### Q-TOF mass spectrometry conditions

Primary and secondary spectra of the samples were collected using an AB Triple TOF 6600 mass spectrometer. The electrospray source conditions after HILIC chromatographic separation were as follows: ion source gas 1 (Gas 1): 60, ion source gas 2 (Gas 2): 60, curtain gas (CUR): 30, source temperature: 600°C, ion capacitor voltage float (ISVF): ± 5500 V (positive and negative two modes); TOF MS scanning range: 60 ~ 1,000 m/z, product ion scanning range: 25 ~ 1,000 m/z, TOF MS scanning cumulative time 0.20 s, and product ion scanning cumulative time 0.05 s. Secondary mass spectra were acquired with information-dependent acquisition (IDA) using a high sensitivity mode, depolymerization potential (DP): ± 60 V (both positive and negative modes), collision energy within 35 ± 15 eV.

#### Metabolomics data processing

Raw data in Wiff format were converted to the mzXML format using the ProteoWizard software, followed by peak alignment, retention time correction, and extraction of peak areas using the XCMS software. The data obtained from XCMS extraction were first subjected to metabolite structure identification, data preprocessing, and then chemometric principles such as PCA, OPLS-DA, and multivariate statistical analysis using SIMCA 14.1 software. Metabolomics data were analyzed by Shanghai Zhongke New Life Biotechnology Co., Ltd.

### Experimental design and animal management

The experiments were performed at the experimental station of the College of Animal Science and Technology, Shihezi University. 240 male yellow-feathered broilers (average initial weight: 189.7 ± 5.9 g) at 14 days were randomly divided into four groups with three replicates of 20 birds each. Every group’s basal diet was supplemented with 0% (group I), 2% (group II), 4% (group III), and 6% (group IV) FGSM. Broilers were maintained in an online flattening model with regular disinfection and cleaning. The initial temperature of the chicken house was 32°C, decreased by 2 ~ 3°C per week to 26°C, with a 24 h light cycle. The experimental period was 42 days and divided into 2 stages, with 14 to 35 days of age in stage 1 and 35 to 56 days of age in stage 2. Chickens were fed and watered freely during the experiment, and immunized according to standard immunization procedures. The chickens used in the experiment were medium-speed yellow-feathered broilers purchased from the Changji Prefecture Green Base Breeding Co. The basal diet consisted of a corn-soybean meal diet, formulated according to the Chinese agricultural industry standard “Nutritional requirements for yellow-feathered broiler chicks” (NY/T 33–2004). The test diet was crushed and turned into powder form. The composition and nutritional levels are shown in Table 1.

**Table 1:** Composition and nutrient levels of diets (%).

Items	14 ~ 35 day				35 ~ 56 day			
	I	II	III	IV	I	II	III	IV
Ingredient (%)								
Corn	59.70	59.80	59.90	60.00	63.50	63.60	63.70	63.80
Soybean meal	27.80	27.70	27.60	27.50	23.00	22.90	22.80	22.70
FGSM	0.00	2.00	4.00	6.00	0.00	2.00	4.00	6.00
UGSM(CON)	6.00	4.00	2.00	0.00	6.00	4.00	2.00	0.00
Vegetable oil	1.50	1.50	1.50	1.50	2.50	2.50	2.50	2.50
Premix	5.00	5.00	5.00	5.00	5.00	5.00	5.00	5.00
Total	100.00	100.00	100.00	100.00	100.00	100.00	100.00	100.00
Nutrient content (%)								
ME (MJ/kg)	11.77	11.78	11.78	11.78	12.16	12.16	12.17	12.17
Crude protein	19.07	19.07	19.07	19.07	17.10	17.10	17.10	17.10
Calcium	0.90	0.90	0.90	0.90	0.80	0.80	0.80	0.80
Available phosphorous	0.40	0.40	0.40	0.40	0.35	0.35	0.35	0.35
Lys	1.13	1.12	1.12	1.12	0.99	0.99	0.99	0.98
Met	0.40	0.40	0.40	0.40	0.34	0.34	0.34	0.34
Thr	0.81	0.80	0.80	0.80	0.73	0.73	0.73	0.72
Ser	0.22	0.22	0.22	0.22	0.19	0.19	0.19	0.19

1. The premix provided the following per kg of diets: Nacl 2.940 g, Lys 0.234 g, Met 1.791 g, VA 8800 IU, VD3 3,000 IU, VE 30 mg, VK2 1.65 mg, VB1 2.5 mg, VB2 6.6 mg, VB3 11 mg, VB4 500 mg, nicotinic acid 60 mg, VB6 4.0 mg, biotin 0.2 mg, folic acid 1.0 mg, VB12 0.02 mg, VC 50 mg, Fe (as ferrous sulfate) 80.0 mg, Cu (as copper sulfate) 8.0 mg, Zn (as zinc sulfate) 60.0 mg, Mn (as manganesesulfate) 70.0 mg, I (as potassium iodide) 0.5 mg, Se (as sodium selenite) 0.3 mg, (14~56 d) Ca 7.415 g, P 2.64 g. 2. The nutritional components were calculated.

### Assessing the growth performance of broilers

The daily feed intake of each group was recorded in detail during the experiment, and the growth performance was calculated in replicates. At days 14, 35, and 56, the broilers were weighed 12 h after fasting, and the leftovers were recovered. The average daily feed intake (ADFI), average daily gain (ADG), and feed conversion ratio (FCR) of the yellow-feathered broilers were calculated.

### Sample collection for broilers

On days 35 and 56 of the experiment, six birds approaching the average weight of each group were weighed and slaughtered individually. Carcass traits, including carcass and abdominal fat, were collected, weighed, and expressed as a percentage of live weight. Blood samples were collected from euthanized birds, placed in non-heparinized tubes, and stored at −20°C until serum biochemistry analysis. Digests from the cecum were collected and divided into two subsamples, stored in RNAse and DNAse-free tubes, placed in liquid nitrogen, and stored at −80°C. One subsample was used for the analysis of intestinal bacteria, and the other was used for the determination of SCFAs.

### Biochemical analysis of broiler serum

Serum biochemical parameters, including total protein (TP), albumin (ALB), blood urea nitrogen (BUN), triglycerides (TG), and total cholesterol (TC) were measured using commercially available kits (Jiancheng Bioengineering Institute, Nanjing, China).

### Analysis of broilers cecal microorganism 16S rRNA

DNA was extracted from the samples using a stool panel DNA extraction kit. After passing the test by 1% agarose gel electrophoresis, the V3 – V4 region of the 16S rDNA of the samples was PCR-amplified using the universal primers 338F (5′-ACTCCTACGGGAGGCAGCAG-3′) and 806R (5′-GGACTACHVGGGTWTCTAAT-3′). The amplification program consisted of: 27 cycles (denaturation at 95°C for 30 s, annealing at 55°C for 30 s, and extension at 72°C for 30 s), and a final extension at 72°C for 10 min. Purified amplified fragments were sequenced using an Illumina MiSeq PE 2500 platform. The double-end sequencing data obtained by MiSeq sequencing removed barcodes and primer splicing, and further removed chimeras and short sequences to obtain clean tags. Under the condition of 97% similarity, the QIIME (v1.8.0) software was used for operational taxonomic unit (OTUs) clustering and species annotation. The mothur software package (version 1.31.2) was used for data processing and analysis.

### Analysis of broilers cecal contents for SCFAs

To analyze SCFA, 0.30 g of the cecal contents was added to 1.5 ml of ultrapure water, vortexed for 30 s, and centrifuged at 5,000*× g* for 4 min. Five hundred μl of supernatant was added to a new sterile Eppendorf tube. Then, 100 μl of 25% metaphosphoric acid solution was added to the tube. The tube was vortexed for 30 s, and centrifuged at 1,500*× g* for 15 min through a 0.22 μm water system. The supernatant was collected into a vial for gas chromatography (Agilent 7890 B) to analyze SCFAs in the sample; the chromatographic column was DB-WAX (30 m × 0.25 mm × 0.50 μm).

### Statistical analysis

One-way analysis of variance (one-way ANOVA) was performed using SPSS 22.0, and significant difference analysis was performed using Duncan’s method (*p* < 0.05). All data are expressed as mean ± SD. A correlation analysis was performed between growth performance, SCFAs, and intestinal microorganisms. Data were entered into SPSS version 22.0, and correlation coefficients were calculated based on Spearman correlation distances. Heat maps were constructed using ORIGIN 2021 to assess the bivariate relationships between variables.

## Results

### The nutritional components of FGSM

After fermentation, the contents of ASP and CP in FGSM reached 0.93 and 11.33%, respectively. It increased 365 and 24.45% with the CON group. The EE content increased to 12.31%. NDF and ADF decreased by 6.82 and 8.23%, compared with those before fermentation. The contents of Ca and P did not change significantly. The free amino acids in FGSM increased compared with CON group, except lysine. The total free amino acids were 823.56 mg/g and 903.84 mg/g in CON and FGSM group, respectively. Among them, glutamate content was the highest, accounting for 22.76 and 22.69% of the total free amino acids (Table 2).

**Table 2:** Chemical composition and amino acid profile of FGSM and CON.

Item	CON	FGSM
Chemical Composition, % of DM		
ASP	0.20	0.93
CP	9.11	11.33
DM	98.30	98.34
ASH	5.09	4.58
EE	10.91	12.31
NDF	60.85	56.70
ADF	48.58	44.58
Ca	0.42	0.47
*P*	0.21	0.20
Amino Acid, %		
Aspartic acid	8.49	7.63
Glutamate	22.76	22.69
Serine	5.15	5.00
Glycine	9.47	9.27
Histidine	2.84	2.92
Arginine	6.24	6.32
Threonine	4.09	4.12
Alanine	5.42	5.67
Proline	6.45	6.79
Tyrosine	1.86	1.98
Valine	5.80	5.93
Methionine	0.38	0.45
Cystine	<0.01	<0.01
Isoleucine	5.00	5.10
Leucine	7.85	8.20
Phenylalanine	4.25	4.36
Lysine	3.96	3.56
Total (mg/g)	823.56	903.84

### Metabolite identification in FGSM

**Figure 1:**
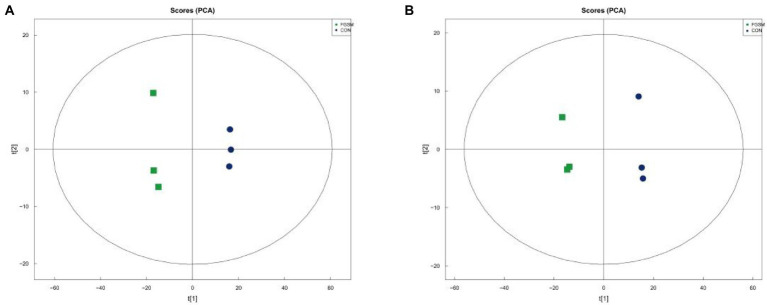
The principal component score plot analysis (PCA) of FGSM. **(A)** PCA plot in positive-ion mode; **(B)** PCA plot in negative-ion mode.

To reveal the changes more intuitively in metabolites after fermentation of grape seed meal, high-resolution LC–MS-based untargeted metabolomics analysis was performed. In the ESI (+) and ESI (−) modes, 240 and 169 ionic features were detected, respectively. Both the ESI positive (Figure 1A, *R*^2^*X* = 0.805) and ESI negative (Figure 1B, *R*^2^*X* = 0.820) models clearly indicated a clear separation trend between FGSM and CON, which reflects the difference in metabolites between the two sample groups. The differentially significant metabolites were identified and screened. A total of 29 differential metabolites (indexed by VIP > 3, *p* < 0.05) were detected in the fermented and unfermented grape seed meal samples. It mainly included 1 metabolite as a nucleic acid, 7 metabolites as lipids, 8 metabolites as carbohydrates, and 5 metabolites as organic acids. In addition, nine unclassified metabolites were identified (Table 3).

**Table 3:** Main difference metabolites between FGSM group and CON group.

Number	Metabolite	Qualification mode	Vip	FC	m/z	rt(S)	*p*-value	Classification
1	Adenosine	Positive	6.07	0.01	268.1	174.04	<0.01	Nucleic acids
2	16-Hydroxypalmitc acid	Positive	5.11	8.86	295.22	214.77	<0.01	Lipids
3	alpha-Linolenic acid	Positive	6.24	1.64	279.23	57.81	0.031	Lipids
4	3-Isopropylmalate	Negative	4.37	0.03	175.06	132.45	<0.01	Lipids
5	Caproic acid	Negative	7.98	0.55	115.08	82.76	<0.01	Lipids
6	Azelaic acid	Negative	10	0.34	187.1	346.41	<0.01	Lipids
7	Linoleic acid	Negative	7.08	0.39	559.47	45.14	0.003	Lipids
8	D-Lactose	Positive	4.09	2.81	360.15	419.43	<0.01	Carbohydrates
9	Maltotriose	Positive	5.83	3.89	522.201	450.16	0.025	Carbohydrates
10	D-Mannose	Negative	5.99	38.05	239.08	308.76	<0.01	Carbohydrates
11	L-Fucose	Negative	4.76	49.49	223.08	253.97	<0.01	Carbohydrates
12	Sucrose	Negative	7.91	0.04	341.11	368.73	0.003	Carbohydrates
13	Tartaric acid	Negative	3.12	0.65	149.01	435.79	0.005	Carbohydrates
14	D-Glucarate	Negative	4.34	1.56	191.02	285.42	0.007	Carbohydrates
15	Ribitol	Negative	4.22	1.41	133.05	195.755	0.049	Carbohydrates
16	L-Phenylalanine	Positive	4.6	5.96	166.08	262.59	<0.01	Organic acids
17	4-Guanidinobutyric acid	Positive	4.59	0.67	146.09	359.45	0.001	Organic acids
18	L-Pyroglutamic acid	Negative	7.32	1.63	128.04	303.51	0.001	Organic acids
19	L-Proline	Negative	3.73	2.07	114.06	315.3	0.001	Organic acids
20	DL-lactate	Negative	4.69	1.75	89.02	233.12	0.007	Organic acids
21	Adenine	Positive/negative	11.89/7.13	4.92/4.81	136.06/134.05	164.87/166.39	<0.01	Unclassified
22	Limonene-1,2-epoxide	Positive	9.27	0.27	213.15	158.73	<0.01	Unclassified
23	Stearidonic Acid	Positive	5.71	0.41	277.22	164.12	<0.01	Unclassified
24	3.alpha.-Mannobiose	Positive	7.41	5.09	325.11	393.60	0.003	Unclassified
25	Scytalone	Negative	6.27	44.88	193.05	160.62	<0.01	Unclassified
26	D-Lyxose	Negative	4.54	5.63	149.05	220.54	<0.01	Unclassified
27	9R,10S-EpOME	Negative	13.02	0.63	295.23	57.60	0.001	Unclassified
28	9-OxoODE	Negative	3.69	0.77	293.21	52.834	0.002	Unclassified
29	9(S)-HODE	Negative	6.00	0.52	295.23	38.738	0.039	Unclassified

m/z is the mass-to-charge ratio; RT is the residence time; VIP means variable importance projection; FC means variable difference multiple, the average value of the peak area obtained by the FGSM group/the average value of the peak area obtained by the UGSM group, the FC value less than 1 indicates that the FGSM metabolite is lower than the UGSM group.

### Analysis of differentially expressed metabolite pathways and enrichment analysis

**Figure 2:**
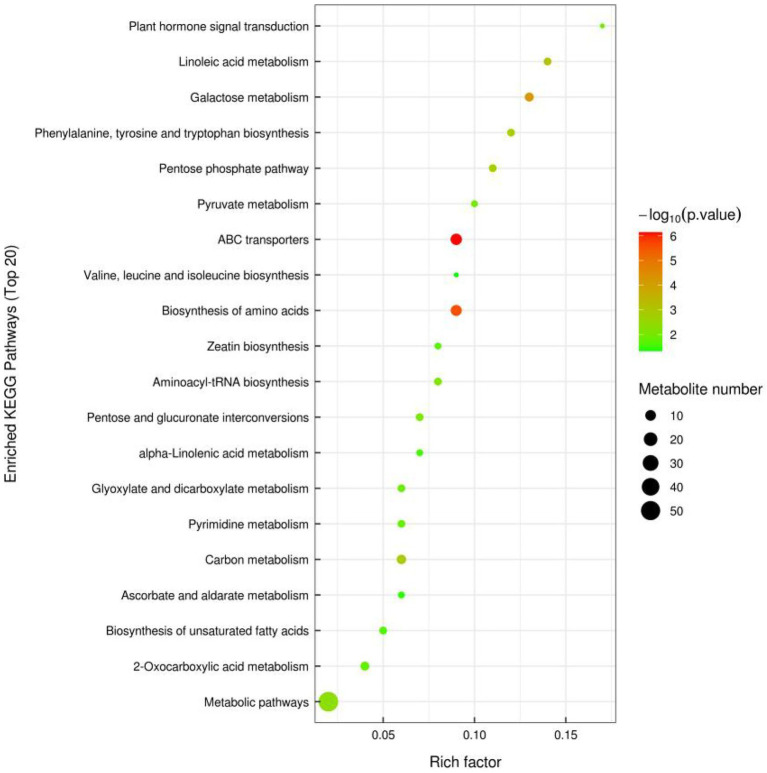
Differentially expressed metabolic pathway maps of FGSM and CON.

In order to observe the significance of differences between metabolic pathways and enrichment of metabolites more intuitively, differential metabolites in the two groups of samples under positive and negative ion mode were combined in this study for enrichment analysis by KEGG pathway. The top 20 metabolic pathways with the highest significance were selected based on *p* values (Figure 2). The pathway enrichment analysis showed that the enriched pathways were mainly involved in Metabolic pathways. The differentially expressed metabolites detected by non-targeted metabolomics were mainly involved in ABC transporters, Biosynthesis of amino acids and Galactose metabolism. Specifically, these metabolic pathways may be critical to identify differences between FGSM and CON, whereas important differentially expressed metabolites play crucial roles in critical pathways.

### Growth performance of broilers

The results showed that different levels of FGSM could increase ADG and ADFI in broilers, and different doses of FGSM had different effects on weight gain in broilers. Compared with group I, ADG was significantly increased and FCR was significantly decreased in groups III and IV (*p* < 0.05). FGSM had a significant effect on ADFI in broilers at the early stage of the experiment. Compared with group I, ADFI was significantly increased in group III, while ADFI was significantly decreased in group IV (*p* < 0.05). We found that the highest ADG and ADFI were observed in group III with 4% FGSM addition. The lowest FCR values were obtained in group IV. These data could all indicate that FGSM improved the growth performance of broilers (Table 4).

**Table 4:** Effects of different levels of FGSM on growth performance.

Items	Group I	Group II	Group III	Group IV
14–35 day				
ADFI/(g/d)	69.47 ± 1.29^b^	68.35 ± 1.78^b^	74.02 ± 3.05^a^	61.99 ± 2.65^c^
ADG/(g/d)	28.40 ± 0.93^b^	31.86 ± 1.45^a^	32.39 ± 1.60^a^	31.48 ± 1.93^a^
FCR	2.45 ± 0.12^a^	2.15 ± 0.14^b^	2.22 ± 0.10^ab^	1.98 ± 0.21^b^
35–56 day				
ADFI/(g/d)	137.04 ± 4.52	131.51 ± 2.62	139.15 ± 8.26	134.60 ± 3.00
ADG/(g/d)	44.46 ± 1.09^b^	44.76 ± 0.56^b^	52.78 ± 1.58^a^	52.39 ± 2.25^a^
FCR	3.08 ± 0.09^a^	2.94 ± 0.05^a^	2.64 ± 0.21^b^	2.57 ± 0.11^b^
14–56 day				
ADFI(g/d)	103.26 ± 1.70^ab^	99.93 ± 1.23^bc^	106.58 ± 2.78^a^	98.30 ± 1.95^c^
ADG/(g/d)	36.43 ± 0.87^c^	38.31 ± 0.97^b^	42.59 ± 0.30^a^	41.93 ± 0.26^a^
FCR	2.83 ± 0.04^a^	2.61 ± 0.07^b^	2.50 ± 0.05^c^	2.34 ± 0.04^d^

In the same line, values with different letter superscripts mean significant differences (*p* < 0.05), while values with the same letter superscripts mean insignificant differences (*p* > 0.05).

### Carcass attributes of broilers

By day 35, compared with group I, the leg muscle rates of groups II, III, and IV were significantly increased by 5.89, 7.94, and 6.70%, respectively (*p* < 0.05). The abdominal fat rate in group IV was significantly lower than that in the other treatment groups (*p* < 0.05). By day 56, the pectoral muscle rate of Group II was significantly higher than other three groups, which were significantly increased by 15.49, 9.35, and 14.06%, respectively (*p* < 0.05). The abdominal fat rate in Group IV was significantly lower than that in Group I by 29.63% (*p* < 0.05) (Table 5).

**Table 5:** Effects of different levels of FGSM on carcass attributes (%).

Items	Group I	Group II	Group III	Group IV
35 day				
Dressed carcass	92.29 ± 1.27	91.51 ± 1.76	92.91 ± 1.44	92.30 ± 0.83
Eviscerated	64.10 ± 0.58	63.62 ± 2.61	63.64 ± 1.97	64.71 ± 1.63
Semi-eviscerate	82.59 ± 0.73	82.41 ± 1.93	82.29 ± 1.65	82.74 ± 1.30
Breast muscle	13.44 ± 1.29	13.64 ± 0.86	13.09 ± 0.99	13.93 ± 0.56
Leg muscle	18.51 ± 0.60^b^	19.60 ± 0.72^a^	19.98 ± 1.07^a^	19.75 ± 1.06^a^
Liver weight	3.71 ± 0.34	3.59 ± 0.30	3.40 ± 0.31	3.58 ± 0.26
Abdominal fat	1.99 ± 0.34^a^	1.98 ± 0.53^a^	2.31 ± 0.40^a^	1.53 ± 0.29^b^
56 day				
Dressed carcass	93.86 ± 1.21	93.52 ± 0.90	93.15 ± 0.65	93.49 ± 0.98
Eviscerated	68.67 ± 1.04	69.28 ± 1.67	69.47 ± 1.55	69.29 ± 1.20
Semi-eviscerate	86.30 ± 1.89	87.48 ± 0.95	86.79 ± 1.14	86.92 ± 1.15
Breast muscle	14.73 ± 1.09^b^	17.43 ± 1.86^a^	15.80 ± 1.00^b^	14.98 ± 1.17^b^
Leg muscle	18.32 ± 0.87	17.76 ± 0.83	18.07 ± 0.63	17.85 ± 0.90
Liver weight	2.62 ± 0.24	2.46 ± 0.36	2.66 ± 0.43	2.77 ± 0.36
Abdominal fat	3.51 ± 0.95^a^	2.96 ± 0.63^ab^	2.83 ± 0.57^ab^	2.47 ± 0.53^b^

In the same line, values with different letter superscripts mean significant differences (*p* < 0.05), while values with the same letter superscripts mean insignificant differences (*p* > 0.05).

### Serum biochemistry of broilers

By day 35, The serum TP level of Group IV was significantly higher than that of Groups I and II (*p* < 0.05). Although Group III was higher than these, it was not significant (*p* > 0.05). By day 56, Groups III and IV were significantly higher than Groups I and II (*p* < 0.05). At 35 and 56 days, the serum TC content of group III was significantly lower than other three groups (*p* < 0.05), and the serum BUN levels of Group I were significantly higher than other three groups (*p* < 0.05). There were no significant differences in the other indicators among the groups (*p* > 0.05) (Table 6).

**Table 6:** Effects of different levels of FGSM on serum biochemical.

Items	Group I	Group II	Group III	Group IV
35 day				
TC/(mmol/L)	2.06 ± 0.77	2.24 ± 0.58	1.98 ± 0.69	2.84 ± 1.02
TG/(mmol/L)	0.67 ± 0.20^a^	0.58 ± 0.13^a^	0.31 ± 0.12^b^	0.79 ± 0.31^a^
TP/(g/L)	28.82 ± 6.49^b^	27.84 ± 2.96^b^	31.13 ± 2.20^ab^	34.35 ± 6.05^a^
ALB/(g/L)	11.34 ± 1.01	10.99 ± 0.92	12.48 ± 2.94	12.99 ± 2.03
BUN/(mmol/L)	1.82 ± 0.71^a^	0.69 ± 0.24^b^	0.54 ± 0.28^b^	0.97 ± 0.48^b^
56 day				
TC/(mmol/L)	2.85 ± 1.34	2.33 ± 0.82	2.12 ± 0.65	2.92 ± 0.63
TG/(mmol/L)	0.57 ± 0.24^a^	0.58 ± 0.11^a^	0.35 ± 0.14^b^	0.58 ± 0.27^a^
TP/(g/L)	23.23 ± 4.30^b^	22.04 ± 5.37^b^	30.36 ± 5.99^a^	29.17 ± 5.87^a^
ALB/(g/L)	9.98 ± 1.83	9.90 ± 1.30	9.90 ± 0.95	10.71 ± 1.06
BUN/(mmol/L)	3.46 ± 0.44^a^	1.09 ± 0.45^c^	1.31 ± 0.74^c^	2.07 ± 0.14^b^

In the same line, values with different letter superscripts mean significant differences (*p* < 0.05), while values with the same letter superscripts mean insignificant differences (*p* > 0.05).

### Out analysis of cecal microbial community in broilers

**Figure 3:**
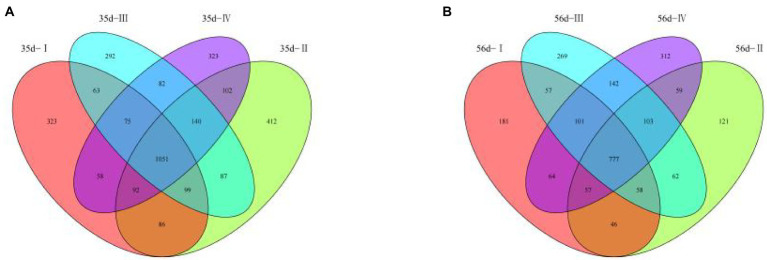
Analysis of Venn Diagram and Dilution Curve of Cecal Microorganism in broilers in different treatment groups. **(A)** Venn diagram for 35-day-old broilers; **(B)** Venn diagram for 56-day-old broilers.

From the 48 samples, 6,010,963 valid reads were obtained after filtering low-quality raw tags and removing chimera sequences. An average number of 125,228 high-quality sequences per sample and an average sequence length of 500 bp for subsequent microbial richness and diversity analysis were obtained. A total of 3,285 OTUs were identified in the cecal contents of 35-day-old broilers, including 1,051 OTUs in the four groups. This accounts for 31.99% of the total OTUs (Figure 3A). In Groups I, II, III, and IV, there were 323, 412, 292, and 323 unique OTUs, respectively. In addition, a total of 2409 OTUs were identified in the cecal contents of 56 day broilers, including 777 OTUs in the four groups, accounting for 32.25% of the total OTUs (Figure 3B). There were 181, 121, 269, and 312 characteristic tables unique to groups I, II, III, and IV, respectively. The results showed that the total number of OUT decreased with the extension of the experimental period, and the number of OUT common to the four groups decreased correspondingly. This indicates that the bacterial diversity in the early experimental period was higher than that in the late experimental period.

### Diversity of cecal microbiota

#### Alpha diversity

Based on Shannon, Simpson, Chao 1 and ACE, which represent the richness and diversity of microbial communities, alpha diversity of microbial communities in the four groups of samples was detected. By day 35, the Shannon and Simpson indices of groups III and IV were significantly higher than those of group I (*p* < 0.05). There was no significant difference in other indices (*p* > 0.05). By day 56, the observed species indices of Groups III and IV were significantly higher than that of Group I (*p* < 0.05); and the other indices were not significantly different (*p* > 0.05). As feeding time increased, the Shannon, Simpson, Chao1, ACE, observed species, and PD whole-tree indices all decreased. The results showed that the addition of FGSM to daily grains affected the abundance and diversity of the gut microbial communities (Table 7).

**Table 7:** Alpha diversity index of broiler cecal microbiome.

Items	Group I	Group II	Group III	Group IV
35 day				
Observed species	831.17 ± 106.07	931.00 ± 79.96	875.50 ± 142.04	909.33 ± 78.41
Shannon	3.85 ± 1.42^b^	4.76 ± 0.90^ab^	5.16 ± 0.76^a^	5.28 ± 0.48^a^
Simpson	0.71 ± 0.24^b^	0.83 ± 0.11^ab^	0.89 ± 0.07^a^	0.91 ± 0.03^a^
Chao1	1010.31 ± 114.44	1116.84 ± 61.67	1065.36 ± 126.28	1093.44 ± 73.22
ACE	1075.79 ± 128.71	1171.34 ± 72.76	1088.34 ± 152.59	1155.05 ± 82.06
Coverage	1.00 ± 0.00	1.00 ± 0.00	1.00 ± 0.00	1.00 ± 0.00
PD_whole_tree	64.63 ± 5.68	65.95 ± 2.33	64.87 ± 6.33	65.58 ± 3.63
56 day				
Observed species	565.00 ± 152.28^ab^	540.17 ± 134.66^b^	715.17 ± 148.07^a^	725.33 ± 109.41^a^
Shannon	3.75 ± 0.58	3.74 ± 0.61	3.82 ± 1.00	4.49 ± 0.49
Simpson	0.80 ± 0.09	0.79 ± 0.11	0.76 ± 0.17	0.86 ± 0.04
Chao1	739.75 ± 170.25	706.63 ± 171.68	903.68 ± 148.77	880.80 ± 145.78
ACE	755.26 ± 169.75	730.93 ± 180.33	937.79 ± 176.91	914.89 ± 156.41
Coverage	1.00 ± 0.00	1.00 ± 0.00	1.00 ± 0.00	1.00 ± 0.00
PD_whole_tree	55.22 ± 8.33	52.27 ± 7.17	60.80 ± 5.17	60.49 ± 6.72

In the same line, values with different letter superscripts mean significant differences (*p* < 0.05), while values with the same letter superscripts mean insignificant differences (*p* > 0.05).

#### Beta diversity

**Figure 4:**
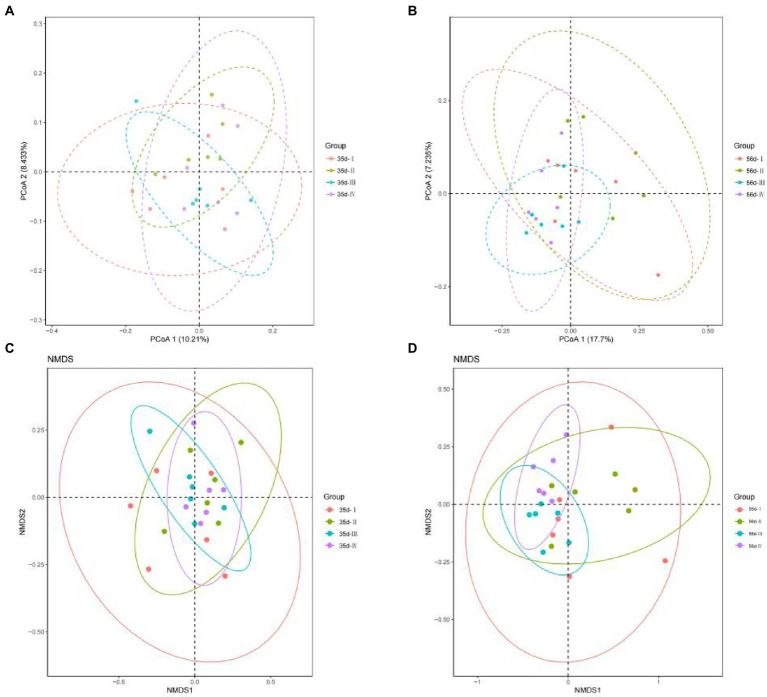
The cecal microbial PCoA map and NMDS map of the unweighted UniFrac distance matrix of broilers in different treatment groups. **(A)** 35 day-PCoA plots; **(B)** 56 day-PCoA plots; **(C)** 35 day-NMDS plots; **(D)** 56 day-NMDS plots.

To facilitate the observation of microbial population differences in the cecal contents between the groups, unweight-based PCoA and NMDS plots were applied to assess beta diversity. By day 35, the contribution rates of principal components 1 and 2 of PCoA were 17.7 and 7.235%, respectively (Figure 4A). By day 56, the contribution rates of principal components 1 and 2 of the PCoA were 10.21 and 8.433%, respectively (Figure 4B). The NMDS plot (Figures 4C,D) shows a clustered distribution of each sample in the four treatment groups, a small difference, similar structure of sample microbiota, and good parallelism. The results showed that the effect of adding FGSM to broiler diets in the early stage was better than that in the late stage, which would lead to a wide distribution of cecal bacteria in broilers in the early stage of the experiment.

#### Variation in cecal microbiota composition

To understand the variation in abundance levels within the cecal samples, we constructed bar graphs of the relative abundance of species. Based on the results of species annotation, we selected the top 10 most abundant species in each sample and grouped them at each taxonomic level (phylum, class, order, family, and genus) to generate column cumulative plots of relative abundances of species, allowing the visualization of relative abundances of species in each sample and their proportions at different taxonomic levels.

**Figure 5:**
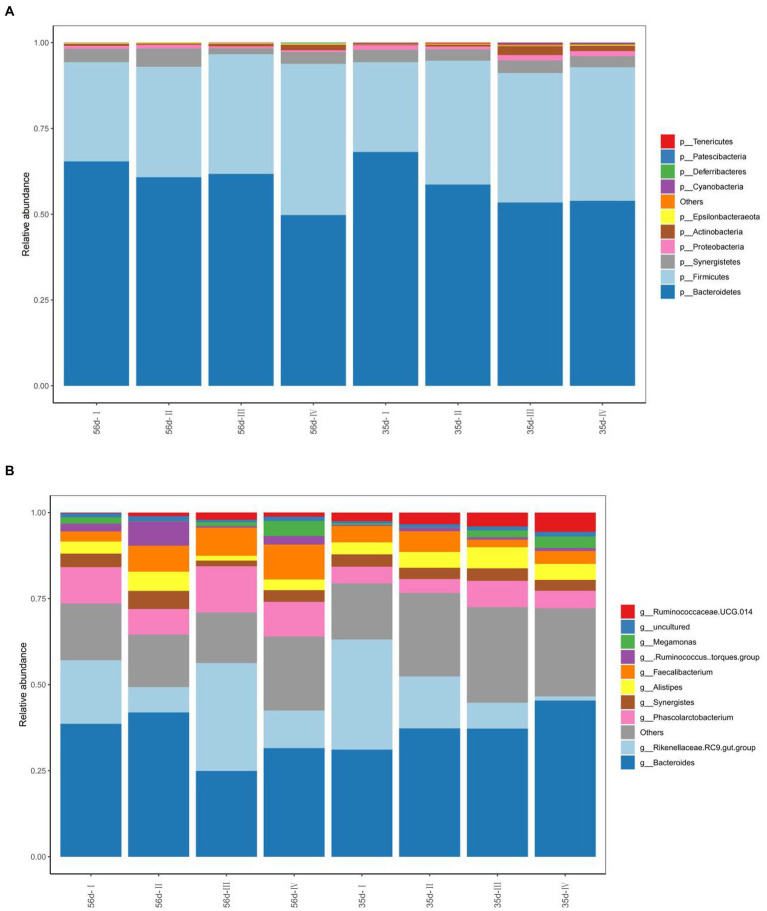
The composition of the cecal microflora of broilers in different treatment groups. **(A)** The phylum level composition of the microflora; **(B)** The genus level composition of the microflora.

At the phylum level, *p_Bacteroidetes*, *p_Firmicutes*, and *p_Synergistetes* were the dominant bacteria, with average abundances of 58.98, 34.87 and 3.49%, respectively. At 35 days, the relative abundance of *p_Bacteroidetes* in Groups III and IV was significantly reduced (*p* < 0.05), and the relative abundance of *p_Firmicutes* was significantly increased (*p* < 0.05) compared with Group I. The relative abundances of *p_Actinobacteria* and *p_Cyanobacteria* in Group III, were significantly higher than those in Group I (*p* < 0.05; Figure 5A). At 56 days, the relative abundances of *p_Actinobacteria* and *p_Firmicutes* in Group IV increased significantly (*p* < 0.05), and the relative abundance of *p_Bacteroidetes* decreased significantly (*p* < 0.05) compared with Group I. The relative abundances of other phyla were not significantly different among the groups (*p* > 0.05).

In addition, the top ten abundant microbial groups at the genus level were obtained through analysis of bacterial composition. They are *g_Bacteroides, g_Rikenellaceae RC9 gut group* and *g_Alitipes* of *Bacteroides*, *g_[Ruminococcus] torques group*, *g_Phascolarctobacterium*, *g_Faecalibacterium*, *g_Ruminococcaceae UCG-014 of p_Firmicutes*, *g_Synergistes*, *g_Megamonas*, uncultured, and others (Figure 5B). The dominant bacterial groups are *g_Bacteroides*, *g_Rikenellaceae RC9 gut group*, and others. At day 35, the relative abundance of *g_[Ruminococcus] torques group* in Groups II and IV increased significantly (*p* < 0.05). The relative abundance of other was significantly increased in Groups II, III, and IV, which were supplemented with FGSM (*p* < 0.05). At 56 days, the relative abundance of *g_Alistipes* in Group II was higher than that in the other test groups.

#### Overview of LEfSe analysis of gut microbiota

Biomarker species with different statistical differences were observed by LEfSe (Linear discriminant analysis Effect Size). Species that met the screening criteria were identified (|LDAscore| > 2), and differential species were colored according to the most abundant group in which the species were located.

**Figure 6:**
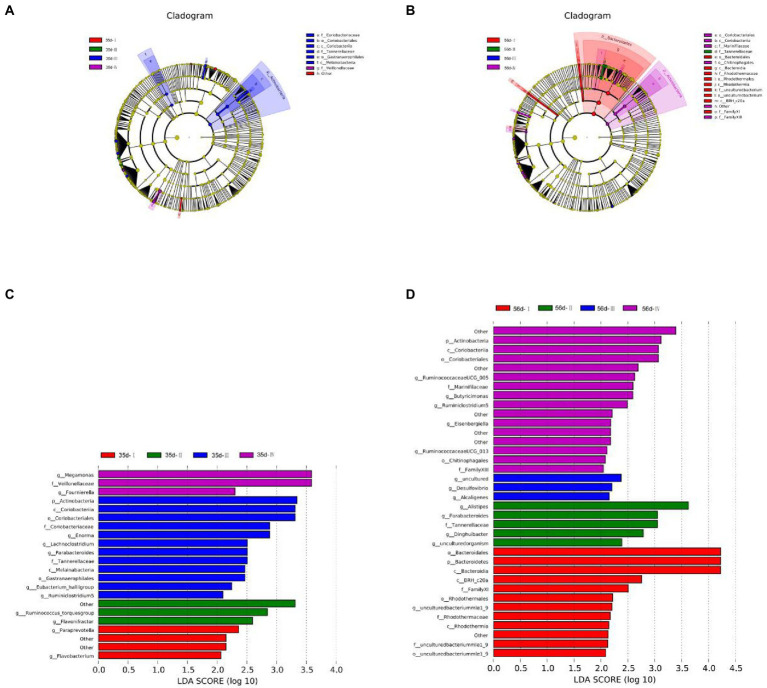
Differential microbial LEfSe analysis and LDA score chart of the cecum of broilers in different treatment groups. **(A)** LEfSe analysis of 35-day-old broilers; **(B)** LEfSe analysis of 56-day-old broilers; **(C)** LDA score chart of 35-day-old broilers; **(D)** LDA score chart of 56-day-old broilers. LEfSe analysis was used to determine the phylogenetic characteristics of specific bacterial taxa and major bacteria in three different groups. Biomarker taxa are highlighted by colored circles and shaded areas. The diameter of each circle is related to the abundance of taxa in the community.

At 35 days, 21 species showed significant differences in abundance between groups, of which Group III showed the highest abundance (12 spieces). The relative abundances of *g_Paraprevotella* and *g_Flavobacterium* were higher in Group I. The dominant bacteria in Group II were *g_Ruminococcus_torques group* and *g_Flavonifractor*. The species with significant differences in Group III were *p_Actinobacteria*, *g_Ruminiclostridium 5*, *g_Eubacterium hallii group*, *g_Lachnoclo -stridium, g_Enorma,* and *g_Parabacteroides*. In Group IV, *g_Megamonas* and *g_Fournierella* were abundant (Figures 6A,C).

At 56 days, 35 species showed significant differences in abundance between groups, with 12 in Group I, 5 in Group II, 3 in Group III, and 16 in Group IV. The relative abundances of *p_Bacteroidetes* and *g_uncultured bacteriummlel_9* in Group I were relatively high. The dominant genera in Group II were *g_Alistipes*, *g_Parabacteroides*, *g_Dinghuibacter*, and *g_uncultured organism*. The species with significant differences in Group III were *g_Desulfovibrio*, *g_Alcaligenes*, and *g_uncultured*. The relative abundances of *p_Actinobacteria*, *g_Ruminococcaceae* -*UCG005*, *g_Butyricimbergiella*, *g_Eisenbergiella*, *g_Ruminiclostridium 5,* and *g_Ruminococcaceae UCG_013* were high in Group IV (Figures 6B,D).

### SCFAs of cecal contents

Acetic acid, propionic acid, butyric acid, and total acid concentrations did not differ among groups II, III, and IV supplemented with FGSM throughout the test period (*p* > 0.05), but their levels were lower than those of group I. In addition, the yield of Butyric acid was increased in group III by day 56 (*p* < 0.05) (Table 8).

**Table 8:** Effects of different levels of FGSM on cecal SCFAs (μg/mL).

Items	Group I	Group II	Group III	Group IV
35 day				
Acetic acid	7.41 ± 0.81	6.25 ± 1.46	6.72 ± 1.64	7.15 ± 1.11
Propionic acid	3.83 ± 0.29	3.42 ± 0.37	3.73 ± 0.62	3.82 ± 0.40
Butyric acid	3.33 ± 0.11	3.08 ± 0.20	3.11 ± 0.24	3.17 ± 0.24
Total acid	14.56 ± 1.11	12.75 ± 1.95	13.85 ± 2.73	13.87 ± 1.48
56 day				
Acetic acid	5.46 ± 0.91	7.16 ± 1.88	6.36 ± 2.37	5.32 ± 1.24
Propionic acid	3.41 ± 0.30	3.91 ± 0.57	3.48 ± 0.55	3.25 ± 0.48
Butyric acid	2.99 ± 0.15^b^	3.11 ± 0.10^b^	3.52 ± 0.41^a^	3.21 ± 0.24^ab^
Total acid	11.87 ± 1.21	14.18 ± 2.47	13.42 ± 3.19	11.78 ± 1.61

In the same line, values with different letter superscripts mean significant differences (P < 0.05), while values with the same letter superscripts mean insignificant differences (P > 0.05).

### Spearman correlation analysis

**Figure 7:**
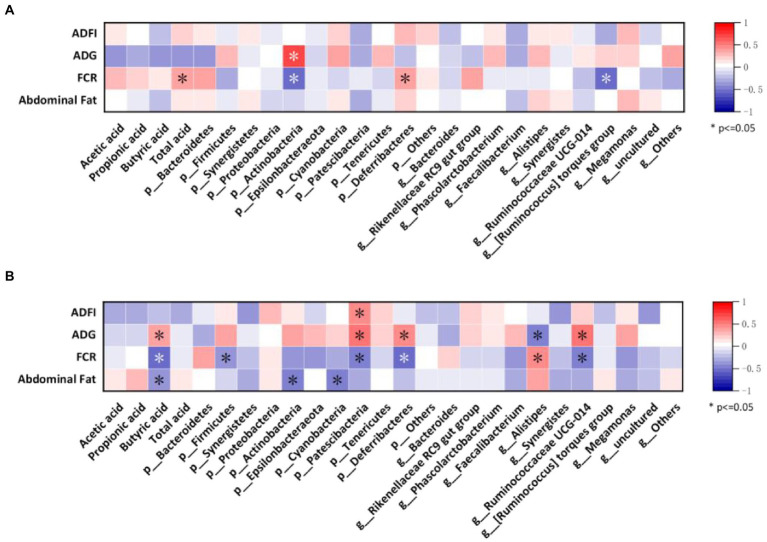
Correlation between the most abundant taxa of the cecal microbiota with growth performance and SCFAs of broilers. **(A)** Heatmap of samples from 35-day-old broilers; **(B)** Heatmap of samples from 56-day-old-broilers.

Finally, we assessed the relationships between growth performance, abdominal fat, gut microbes, and SCFAs using Spearman’s correlation analysis. As shown in Figure 7A, at 35 days, FCR of broilers was positively correlated with total acid content and *p_Deferribacteres*, and ADG was positively correlated with *p_Actinobacteria* while showing the opposite trend with FCR. Meanwhile, FCR also showed a negative correlation with *g_[Ruminococcus] torques group*.

Figure 7B shows the correlation between growth performance, abdominal fat with gut microbes, and SCFAs in broilers at 56 days. ADFI was found to be positively correlated with the *p_Patescibacteria*. Additionally, ADG was positively correlated with butyric acid, *p_Patescibacteria*, *p_Deferribacteres*, and *g_Ruminococcaceae UCG-014* and negatively correlated with *g_Alistipes*. FCR showed a negative correlation with butyric acid, *p_Firmicutes*, *p_Patescibacteria*, *p_Deferribacteres*, and *g_Ruminococcaceae UCG-014* and a positive correlation with *g_Alistipes*. Moreover, abdominal fat was negatively correlated with butyric acid, *p_Actinobacteria*, and *p_Cyanaceae.*

## Discussion

### Improving feed nutrient content to increase broiler feed intake

Fermented grape seed meal by Bacteria coupled fermentation with enzymes has advantages over the individual processes of probiotic fermentation and protease enzymatic hydrolysis. Acid protease can improve the nutritional quality of grape seed meal. By directly increasing the number of proteases, the reaction rate of protease hydrolysis is increased. The macromolecular protein in feed material is decomposed into peptides and free amino acid, so as to improve the conversion rate of feed in animal organism. When fermenting grape seed meal with *Saccharomyces cerevisiae*, the acid-soluble protein content increases for the following reasons: yeast cells contain a large number of proteins and amino acids. And, yeast can produce protease during the fermentation process. As fermentation proceeds, nutrient-active substances such as organic acids (tartaric, malic, and citric acid) are produced during yeast growth and reproduction. They involved in enzymatic reactions through the tricarboxylic acid cycle, allowing proteases to break down large molecules of proteins at a faster rate. Also, microorganisms can convert non-protein nitrogen into protein nitrogen, which can be absorbed and used by livestock (Tchorbanov and Lazarova, 1988; Zhang, 2012; Wu, 2018). Moreover, the contents of NDF and ADF were reduced. The main reason was that during the fermentation process, the microorganisms produced cellulases that could decompose cellulose, hemicellulose and lignin during growth and reproduction, which reduced the fiber content in the FGSM (Rodríguez Muela et al., 2017). Simultaneously, organic acids produced by *S. cerevisiae* during fermentation could improve feed palatability. The relative concentration of monosaccharides used as sweeteners in feed such as D-mannose and L-fucose increased in FGSM. It has a unique volatile aroma of grapes, fragrant taste, and good palatability. Therefore, the fermented grape seed meal by Bacteria coupled fermentation with enzymes can effectively improve the nutritional composition of feed ingredients and improve animal feed intake.

### FGSM promotes broiler growth and improves carcass quality

In this study, the addition of FGSM significantly increased broiler growth performance. The growth performance of animals is influenced by muscle proteins, which constitute the net balance of protein synthesis and degradation. Amino acids are basic components of proteins and play roles in regulating nutrient transport, gut microbiota, and antioxidant responses (Mott et al., 2008). In the intestine, proteins are broken down into small peptides and free amino acids by enzymes and transported through different nutrient transporters (Wu et al., 2014). By untargeted metabolomics analysis, we found that the relative concentration of adenosine was lower in FGSM. Adenosine plays an important role in cellular metabolism by acting as an intermediate in ATP synthesis. Studies have shown that phosphoribose in yeast cells is degraded into a mixture of adenosine, adenine, ribose, single nucleotide, and polynucleotides by an enzymatic system. Then Adenosine and other substances are phosphorylated through the pathway of glycolysis to produce ATP, which then binds to the protein hydrolysis region of ABC transporters, causing ATP to be hydrolyzed. The energy generated by metabolism changes the conformation of transporters, resulting in transporters transporting various sugars, amino acids, proteins and other cellular metabolites to the extracellular space (Guerrero and Mullet, 1986). It means that the content of small peptides and free amino acids increases through this metabolic pathway. This is supported by the results of the free amino acid content in FGSM. Therefore, enhanced intestinal uptake of amino acids in broilers fed diets supplemented with FGSM would favor protein deposition and ultimately promote growth.

In the early part of the trial, the breast and leg muscle rates increased in broilers that were fed FGSM, but there was no significant difference between the full clearance rate and half clearance rate. This suggests that broiler muscle protein metabolism had the potential to change (Lee et al., 2009). Rodas et al. (1995) found that enhanced synthesis of metabolic proteins can be observed when TP levels increase. The results of this study showed that the addition of 6% FGSM could significantly increase the TP content in the serum of broilers and increase the ALB content. This indicates that FGSM could improve serum TP and ALB concentrations and enhance protein metabolism in broilers. Dhanalakshmi et al. (2007) showed that good nutritional status maintained high levels of total serum protein and albumin, with increased levels of both indicating high metabolic activity. This is similar to (Feng et al., 2007), which demonstrate the positive effect of fermented feed on the metabolic capacity of broilers. BUN is one of the protein metabolites, and its content reflects the status between protein metabolism level and amino acid balance in animals to some extent (Scott et al., 1982). When serum BUN levels are reduced, amino acid catabolism decreases and nitrogen deposition in the body increases, indicating enhanced protein metabolism (Sugiharto et al., 2020). Therefore, adding FGSM to the diet plays an important role in promoting the absorption and utilization of protein in broilers.

At the same time, we found that FCR gradually decreased as the amount of FGSM added increased. In particular, the FCR of group IV was significantly lower than that of the remaining three groups, indicating that the addition of FGSM to the diets had better economic benefits. One study shown that the addition of peas fermented with probiotics or treated with complex enzymes to broiler diets reduces FCR throughout the experimental period (Goodarzi et al., 2017). In another study, the addition of fermented corn gluten flour to daily food improved ADG in broilers (Yan et al., 2018). This is more reflective of the improved nutritional composition of the feed after fermentation or enzymatic digestion, which has the effect of promoting animal growth.

### FGSM regulates lipid metabolism in broilers

Fat deposition in broilers is dominated by three areas: subcutaneous fat, abdominal fat, and intramuscular fat, with abdominal fat receiving more attention than other adipose tissues. Abdominal fat becomes visible at 7 days of age in broilers (Kim and Voy, 2021), indicating a rapid growth rate. The number of adipocytes gradually increases with age and broiler fat cells increase in size as they absorb and store fatty acids (Bai et al., 2015). Because of ease in dissection and weighing, abdominal fat percentage is widely used to assess the total lipid content in broilers. In the group fed with 6% FGSM, there was a significant reduction in abdominal fat percentage and serum TG levels, suggesting that FGSM regulates body lipid metabolism (Yasar and Yegen, 2017).

After fermentation, the number of probiotic bacteria in FGSM increased. Homma and Shinohara (2004) indicated that probiotics can inhibit lipid synthesis and promote fatty acid catabolism, thereby inhibiting abdominal fat deposition. TC and TG are important components of blood fat, and their levels reflect the absorption and metabolism of fat in the body. Kim and Voy (2021) reported that abdominal fat is the main site of TG storage in mature broilers. When the weight of abdominal fat in broilers decreased, the TG content stored in abdominal fat also decreased and the TG content in the serum increased. Our study found that the addition of appropriate FGSM concentrations resulted in a decrease in serum TG levels. Ooi and Liong (2010) reported that some probiotics are effective at lower levels, whereas others require higher amounts to exert cholesterol-lowering effects. Cholesterol adsorbed in the cell wall of probiotics, or the cells of the bacterium can absorb cholesterol and allow cholesterol removal, thus promoting fat metabolism (Milićević et al., 2014).

The oils in grape seed meal are rich in fatty acids (FAs). Compared with unsaturated fatty acids, saturated fatty acids are more stable. However, they can be converted from saturated fats (palmitoleic and stearic acids) to unsaturated fatty acids (oleic, linolenic, and free fatty acids) by microorganisms with a range of active proteins, such as dehydrogenases and lengthening enzymes (Rhead et al., 1971). The unsaturated fatty acids present in FGSM reduce fat accumulation (Riera-Heredia et al., 2020). Polyunsaturated fatty acids (PUFAs) are classified as n-3 or n-6 depending on the position of the first double bond relative to the methyl end of the molecule. At the cellular level, Fleckenstein-Elsen et al. (2016) found that n-3 PUFAs inhibit lipid production and attenuate lipid accumulation in adipocytes. In contrast, n-6 PUFAs tended to be pro-lipogenic (Massiera et al., 2003). According to the results of non-targeted metabolomics, α-linolenic acid belonging to n-3 PUFAs was upregulated and linoleic acid belonging to n-6 PUFAs was downregulated, resulting in the inhibition of abdominal fat deposition.

### FGSM alters the composition of broilers intestinal bacteria

Intestinal microorganisms play important roles in maintaining animal health, growth, feed absorption, and utilization (Zhang et al., 2018). However, its composition and structure are constrained by various factors such as feed, age, and feeding method (Carrasco et al., 2019). Among these, feed has the greatest effect on intestinal bacteria, which changes their structure and quantity. The health of animals is affected by the production of specific metabolites through biological reactions (Doré and Blottière, 2015). Therefore, changes in feed nutrient composition are very important for the regulation of intestinal bacteria. We found that organic acids, monosaccharides, and unsaturated fatty acids, which are differential metabolites produced by FGSM, have some regulatory effects on broiler gut microbiota.

Our study showed that dietary supplementation of FGSM altered the composition of the cecal microbial community and increased cecal microbial diversity in broilers. At phylum level, Bacteroides, Firmicutes, Synergistetes, and Proteobacteria were the four dominant groups in broiler cecal microorganisms. Similar to the results of Ma et al. (2018), but the third dominant phylum was altered. *Synergistetes* is a newly discovered genus of bacteria that is widely found in anaerobic environments such as the animal intestines and soil (Jumas-Bilak et al., 2009). As a class of Gram-negative, obligate anaerobic bacteria, most of them can grow at room temperature and neutrally and have the ability to degrade amino acids. Simultaneously, *Synergistetes* caused changes in the dominant flora in the gut of broilers, which was responsible for the changes in the structure of the gut community.

When the diet contains high levels of free amino acids, unsaturated fatty acids, and monosaccharides, intestinal bacteria in the body have a higher proportion of *Firmicutes* and a lower proportion of *Bacteroidetes* (De Filippo et al., 2010). Both have a direct relationship in improving the digestion and absorption of nutrients and energy metabolism in animals (Jumpertz et al., 2011), both induce fat deposition in animals. However, *Firmicutes* more effectively extract energy from food, thereby promoting weight gain. Additionally, most studies support an increased (F/B) ratio in obese individuals (Crovesy et al., 2020). In this study, the (F/B) ratio was <1 in all four test groups, and the (F/B) ratio also increased correspondingly with the increase of FGSM addition, but the deposition of abdominal fat decreased. In a Ukrainian study, individuals with an F/B ratio ≥1 were 23% more likely to be overweight than those with an F/B ratio <1 (Koliada et al., 2017). Therefore, an increase in the (F/B) ratio cannot be used as the sole basis for obesity, and the specific ratio has to be discussed. Bervoets et al. (2013) reported that a higher (F/B) indicates a relative abundance of energy-metabolizing microorganisms in the gut microbiota. Similarly, an increased F/B ratio is often associated with growth-promoting performance (Salaheen et al., 2017). In this study, the addition of different concentrations of FGSM to the diet did not change the status of *Bacteroidetes* as the dominant bacterial group. However, with increasing FGSM levels, the relative abundance of *Bacteroidetes* decreased and *Firmicutes* increased. The reduction in abdominal fat percentage, increase in daily weight gain, and reduction in FCR in broilers provided more evidence of efficient energy utilization.

*Actinobacteria* are key players in the maintenance of the intestinal barrier and consist of three major families of anaerobic bacteria (*Bifidobacteria*, *Propionibacterium*, and *Corynebacterium*) and one family of aerobic bacteria (*Streptomyces*). Among these, *Bifidobacteria* are considered important probiotics in *Actinobacteria*. Studies have reported that most antimicrobials are composed of bioactive substances produced by *Actinobacteria* and show good antibacterial properties in controlling pathogenic microorganisms (Gomes et al., 2017). The results of the non-targeted metabolome showed an increase in the relative concentrations of sugar metabolites such as D-mannose and L-amylose. When animals consume diets containing monosaccharides, they benefit from increased abundance of *Bifidobacteria* and colonization of the intestinal tract, thereby improving broiler intestinal health (Dev et al., 2020). Previous studies have shown that *Actinobacteria* can utilize complex carbohydrates and contribute to improved growth performance and increased feed conversion efficiency in broilers (Courtin et al., 2008). Also, the relative abundance of *Bifidobacteria* has been correlated with fat deposition. In a study comparing fecal samples from lean and obese individuals, it was found that the abundance of *Bifidobacteria* differed between the two, their relative abundance showed a negative correlation with the percentage of body fat (Teixeira et al., 2013).

To further investigate the effect of FGSM on gut microbiota diversity in yellow-feathered broilers, we analyzed differences at the genus level. Among the four groups of samples, *Bacteroides* and *Rikenellaceae RC9* gut *group* under *p_Bacteroides* were the dominant genera. This genus help the host to break down polysaccharides, generate volatile fatty acids and butyrate. In this way, it improves the host immunity and facilitating the regulation of the intestinal immune system (Zhang et al., 2019). In contrast, [*Ruminococcus*] *torques group*, *Phascolarcto -bacterium*, *Faecalibacterium*, and *Ruminococcaceae UCG-014* under *p_Firmicutes* (Louis and Flint, 2017) which can increase the concentration of SCFAs in the intestine. Macfarlane and Macfarlane (2012) pointed out that the content of SCFAs in the gut is closely related to the composition and abundance of gut microbiota. So FGSM increases the concentration of SCFAs in the gut. The main sources of SCFAs in the gut of animals are bacterial metabolites produced by anaerobic fermentation of carbohydrates that are not easily digested (Garron and Henrissat, 2019). It not only acts as an energy source for host cells and gut microbiota, but also improves host lipid and glucose metabolism. Moreover, the production of acetic acid and butyric acid by gut microbiota can cause an increase in broiler feed intake (Chen et al., 2022). Therefore, the pH in the intestine of broilers fed with FGSM decreased, which inhibited the growth of harmful microorganisms. Thereby improving the structure of the intestinal flora and enhancing growth performance, further exerting a potential promotional effect on intestinal health.

In summary, the nutritional composition of grape seeds changed after fermentation, and the concentrations of free amino acids, unsaturated fatty acids, monosaccharides, and organic acids increased. Adding 4% ~ 6% FGSM to broiler diets promotes broiler growth and regulates fat metabolism by altering gut microbiota structure.

## Data availability statement

The datasets presented in this study can be found in online repositories. The names of the repository/repositories and accession number(s) can be found at: https://www.ncbi.nlm.nih.gov/, PRJNA858635.

## Ethics statement

The animal study was reviewed and approved by Animal Ethics Committee of the First Affiliated Hospital of Shihezi University School of Medicine. Written informed consent was obtained from the owners for the participation of their animals in this study.

## Author contributions

SN and CN carried out the experimental design of the study. SN, MY, XZ, and HW contributed to the experimental implementation. SN, MY, HW, and JL contributed to the sampling for this study. SN, MY, and CN contributed to data analysis. SN, MY, XZ, JN, CC, WZ, and CN contributed to the writing of the article. All authors contributed to the article and approved the submitted version.

## Funding

This work was supported by the Science and Technology Innovation Talent Program of Xinjiang Production and Construction Corps (project no. 2020CB023) and the Young Innovation Talent Cultivation Program of Shihezi University (project no. CXRC201807).

## Conflict of interest

The authors declare that the research was conducted in the absence of any commercial or financial relationships that could be construed as a potential conflict of interest.

## Publisher’s note

All claims expressed in this article are solely those of the authors and do not necessarily represent those of their affiliated organizations, or those of the publisher, the editors and the reviewers. Any product that may be evaluated in this article, or claim that may be made by its manufacturer, is not guaranteed or endorsed by the publisher.
